# Focus on photosynthesis

**DOI:** 10.1093/plcell/koae204

**Published:** 2024-07-20

**Authors:** Nancy A Eckardt, Ralph Bock, Roberta Croce, J Clark Lagarias, Sabeeha S Merchant, Kevin Redding

**Affiliations:** The Plant Cell, American Society of Plant Biologists, USA; The Plant Cell, American Society of Plant Biologists, USA; Max Planck Institute of Molecular Plant Physiology, Potsdam-Golm 14476, Germany; The Plant Cell, American Society of Plant Biologists, USA; Department of Physics and Astronomy, Faculty of Science, Vrije Universiteit Amsterdam, Amsterdam 1081 HV, The Netherlands; The Plant Cell, American Society of Plant Biologists, USA; Department of Molecular and Cellular Biology, University of California, Davis, CA 95616, USA; The Plant Cell, American Society of Plant Biologists, USA; Department of Plant and Microbial Biology, University of California, Berkeley, CA 94720, USA; The Plant Cell, American Society of Plant Biologists, USA; Center for Bioenergy and Photosynthesis, School of Molecular Sciences, Arizona State University, Tempe, AZ 85287, USA

Photosynthesis encompasses the processes by which plants, algae, and photosynthetic bacteria transform light energy into chemical energy and fix carbon from the atmosphere into organic compounds to support their growth and development. We cannot overstate the importance of photosynthesis to life on Earth. Yet, critical gaps remain in our understanding of photosynthesis, its interplay with other metabolic pathways, and the adaptation and acclimation processes that allow photosynthetic organisms to live almost everywhere on our planet. Filling these gaps will enable improving the efficiency of photosynthesis in crop plants, accurately modeling the global carbon cycle, and enhancing terrestrial carbon sequestration to combat global climate change.

This focus issue includes 2 commentaries, a perspective, 6 in-depth review articles, and 8 research articles. The commentary by Loudya et al. (2024) discusses plastid retrograde signaling: the nature of plastid-derived signals that regulate differentiation of photosynthetic cells and the role of the enigmatic protein GUN1. Two articles present a series of minichapters or vignettes written by experts in their areas of research. The commentary by Eckardt et al. (2024) addresses 11 open questions in photosynthesis research, and a companion perspective from Croce et al. (2024) presents 12 vignettes on improving photosynthesis to increase crop yields.

These are followed by 6 in-depth review articles that cover aspects of the major protein complexes participating in the photosynthetic light reactions. (i) Bryant and Gisriel (2024) describe progress in the structural resolution of light-harvesting phycobilisomes and chlorophyll-binding proteins of cyanobacteria, red algae, and cryptophytes. (ii) Ostermeier et al. (2024) review the structure, biogenesis, and evolution of thylakoid membranes. (iii) Rolo et al. (2024b) review the structure, function, and assembly of vascular plant photosystem (PS) I, and (iv) Komenda et al. (2024) cover the biogenesis and maintenance of PSII. (v) Rühle et al. (2024) cover the structure and function of chloroplast ATP synthase, including recent engineering strategies related to light-driven nanomotors. (vi) Finally, Degen and Johnson (2024) review photosynthetic control at the cytochrome (cyt) *b*_6_*f* complex, a protective mechanism that prevents light-induced damage to PSI.

Eight research articles present exciting new results in a range of topics in photosynthesis research. (i) Rojas et al. (2024) used a synthetic pentatricopeptide repeat protein to show that HIGH CHLOROPHYLL FLUORESCENCE 173 (HCF173) is required for light regulation of *psbA*, which encodes the PSII reaction center protein D1. They further report that HCF173 activates *psbA* translation by sequestering the RNA it binds to maintain an accessible ribosome binding region. (ii) Rolo et al. (2024a) report on a new PSI assembly factor, CO-EXPRESSED WITH PSI ASSEMBLY1, and show that it plays a nonessential but important role in PSI assembly. (iii) Xin et al. (2024) report the cryo-electron microscopy structure of alternative complex III (ACIII) from the anoxygenic phototrophic bacterium *Chloroflexus aurantiacus*. ACIII complexes functionally replace cyt *bc*_1_/*b*_6_*f* complexes in many photosynthetic bacteria; thus, this study improves our understanding of photosynthetic electron transport. (iv) Peltier et al. (2024) expand upon previous findings about alternative photosynthetic electron transfer pathways by quantifying the contribution of cyclic, pseudocyclic, and chloroplast-to-mitochondria electron flows to net photosynthesis in *Chlamydomonas reinhardtii*. (v) Riché et al. (2024) investigated state transitions, the process that balances excitation energy between PSI and PSII, highlighting the role of the cyt *b*_6_*f* C-terminus in the interaction with the STT7 kinase in the regulation of state transitions. (vi) Che et al. (2024) investigated the Arabidopsis (*Arabidopsis thaliana*) mutant *resistance to Phytophthora1* (*rph1*) and show that RPH1 is a conserved intrinsic thylakoid protein that promotes the efficient assembly of cyt *b_559_* during early PSII assembly. (vii) Penzler et al. (2024) conducted a *proton gradient regulation5* (*pgr5*) suppressor screen and identified mutations affecting 12 photosynthesis-related proteins, including a cyt *b*_6_*f* assembly factor and a factor linking PGR5 to cyt *b*_6_*f* stability. (viii) Finally, Groot Crego et al. (2024) uncover gene family expansion as a genomic driver of Crassulacean acid metabolism evolution in the genus *Tillandsia* (Bromeliaceae).

We encourage authors to continue to submit their best work in photosynthesis research to *The Plant Cell*. Articles on topics related to photosynthesis published within 8 to 12 months of this focus issue will be added to an online collection.
